# The Influence of Individualization in External Load Control on Anaerobic Performance in a Women’s Soccer Team

**DOI:** 10.3390/sports14040138

**Published:** 2026-04-01

**Authors:** Alexandre Galvão da Silva, Caroline Cavalcanti de Freitas, Alef Serrat Pinheiro, Débora Dias Ferraretto Moura Rocco, Caroline Simões Teixeira, Luis Alberto Rosan, Rodrigo Kallás Zogaib

**Affiliations:** 1 Clube de Futebol Profissional, Santos Futebol Clube, 11075-500 Santos, SP, Brazil; carolfreitaas230900@gmail.com (C.C.d.F.); alefserrat@gmail.com (A.S.P.); luizalbertorosan54@gmail.com (L.A.R.); rodrigozogaib@gmail.com (R.K.Z.); 2 Faculdade de Educação Física, Universidade Santa Cecília, 11045-100 Santos, SP, Brazil; drocco@unisanta.br (D.D.F.M.R.); carolteixeira@unisanta.br (C.S.T.)

**Keywords:** athletes, periodization, performance

## Abstract

Soccer is an intermittent sport that requires complex and well-adjusted physiological responses from athletes. The training load allows athletes to optimize physical adaptations and reduces the risk of musculoskeletal injuries. In women’s soccer, the implementation of load control and individualization strategies has shown promise for enhancing anaerobic performance and injury prevention. This study aimed to compare the performance levels of professional women’s soccer players before and after the implementation of relative external load (RELC) for training prescription. Twenty-seven female professional soccer athletes (mean age 29.4 ± 6.2 years) were evaluated. Metrics such as total distance, sprint distance, number of sprints, accelerations, and decelerations were collected using the GPS-based device Catapult One (Catapult). Athletes were assessed in two games, with 6 months’ difference between matches: Game 1, without RELC implementation, and Game 2, with RELC. Significant differences were found between both periods. Sprint distance increased from 391 m to 450 m (+15%, d = 0.49, *p* ≤ 0.05), and sprint count increased from 14 to 17 (+21%, d = 0.35, *p* ≤ 0.05), showing improved performance related to increased physical output in the second half of the season. These findings suggest potential performance improvements associated with individualized load control over the course of the season.

## 1. Introduction

Soccer is an intermittent sport characterized by high-intensity actions such as sprints, changes of direction, accelerations, and decelerations, which require complex and well-adjusted physiological responses from athletes [1,2]. To achieve supercompensation and sustained performance improvement, training stimuli must be applied based on rigorous load control, respecting the principles of progressive overload, specificity, and biological individuality [3].

Training load control is one of the main pillars of exercise physiology applied to elite sports. It allows for the monitoring and adjustment of intensity, volume, and frequency of stimuli imposed on the body, aiming to optimize physical adaptations and reduce the risk of musculoskeletal injuries caused by training overload [4,5]. Load can be classified into two main domains: external load and internal load. External load refers to the objective and quantifiable physical work performed by an athlete during training or competition. It encompasses variables that describe the volume and intensity of movement. Common metrics used to monitor external load include total distance covered, distance covered at high speed, the number and distance of sprints, and the number of accelerations and decelerations. On the other hand, internal load represents the physiological responses of the athlete to the external training stimulus. It reflects how the body perceives and reacts to the imposed workload and is crucial for understanding individual adaptations and fatigue. Typical metrics for internal load monitoring include heart rate (HR) and heart rate variability (HRV), blood lactate concentration, and the rate of perceived exertion (RPE) [6,7,8]. Both external and internal loads are essential for a comprehensive understanding of an athlete’s training status. Monitoring only one domain can provide an incomplete picture, as athletes with similar external loads may present very different internal responses. Therefore, integrating both types of load measurements allows for a more precise individualization of training, optimizing performance while minimizing the risk of overreaching or overtraining [1].

Recent investigations have emphasized the importance of individualized external load prescription as a critical strategy to enhance the specificity and effectiveness of training interventions, particularly in team sports characterized by high mechanical demand. Load control can help ensure optimal stimuli that lead athletes to their peak performance but without generating external overload that increases the risk of injuries [9,10]. In this context, the application of personalized velocity thresholds—such as maximal sprinting speed (MSS) or maximal aerobic speed (MAS)—has emerged as a key methodological refinement over fixed thresholds (e.g., 19 or 23 km/h), especially for female athletes whose neuromuscular and physiological profiles differ from their male counterparts [11,12]. When athletes are trained based on individualized intensity zones, sprint and acceleration efforts are more precisely calibrated to elicit meaningful adaptations in anaerobic capacity, speed reserve, and repeated sprint ability.

The use of GPS tracking devices in team sports like soccer has enabled significant advances in quantifying external load and designing more precise and effective training programs [9,10]. According to Negrão et al. [3], load individualization is crucial for training effectiveness, especially in high-performance athletes, and must consider not only the athlete’s historical performance but also their current level of physical and biological preparedness [11].

Moreover, the growing availability of wearable technologies and automated tracking systems has allowed researchers and practitioners to monitor external load fluctuations with greater granularity, fostering advances in microcycle design and periodization [13,14]. Karlsson et al. [15] demonstrated that the longitudinal quantification of external load, considering positional demands and menstrual cycle phases in women’s soccer can significantly improve training responsiveness and reduce cumulative fatigue. These findings underscore the need to contextualize load monitoring within the unique physiological and biomechanical profiles of female players, advocating for sex-specific approaches in elite performance programs [16].

Female athletes present unique physiological and hormonal characteristics that influence their response to training and recovery processes. Factors such as the menstrual cycle, fluctuations in estrogen and progesterone levels, and differences in muscle composition and metabolism can affect performance, fatigue, and injury risk. Therefore, precise load monitoring is particularly important in female athletes to individualize training programs that respect these physiological variations. The proper control of training load helps optimize adaptations while minimizing risks related to hormonal fluctuations. Understanding and integrating these sex-specific factors in load management is crucial for maximizing performance and health in female athletes [17].

In recent years, external load control has emerged not only as a preventive strategy but also as a crucial factor in enhancing performance metrics in soccer. Monitoring metrics such as sprint distance, high-intensity running, and deceleration events allows for precision in training periodization, which has been shown to improve repeated sprint ability, anaerobic capacity, and match readiness [18,19].

Although studies on women’s soccer remain limited, findings from elite male athletes support that individualized load management positively correlates with performance outcomes such as sprint ability, the high-speed distance covered, and neuromuscular readiness [20]. These principles are increasingly adopted in women’s sport to promote similar performance benefits.

Furthermore, recent reviews advocate for a dual-load monitoring approach that integrates external (e.g., sprint distance, accelerations) and internal (e.g., session-RPE, HRV) markers to assess the comprehensive physiological cost of training [1,19,20]. While the present study focuses primarily on external load metrics, the literature supports the theory that the interplay between mechanical and perceptual demands influences fatigue accumulation, recovery kinetics, and training efficiency. Consequently, when individualized external load prescriptions are combined with internal feedback, practitioners can generate holistic training models that optimize performance output while mitigating injury risk [21].

In women’s soccer, still underrepresented in research compared to men’s soccer, the implementation of load control and individualization strategies has shown promising results in enhancing anaerobic performance and injury prevention [12,13]. Thus, it is important to understand the practical applicability of this data produced by the GPS to the professional women’s soccer context, linking individualized external load prescription to its effects on physical game performance. Therefore, the objective of this study was to compare the physical performance levels of professional women’s soccer players before and after the implementation of relative external load control (RELC) as a strategy for training prescription and monitoring. Unlike a randomized or controlled experimental intervention, this study employed a retrospective observational design, based on data collected from competitive matches. The comparison aimed to assess whether the systematic application of individualized external load targets, derived from athlete-specific match performance metrics, was associated with improvements in anaerobic performance indicators, such as sprint distance and number of high-speed actions, over the course of the competitive season.

## 2. Materials and Methods

### 2.1. Participants

Twenty-seven professional female soccer athletes were evaluated, with a mean age of 29.4 ± 6.2 years. The twenty-seven athletes from the professional team who were fit (without injuries or absences) and actively participating in the competitive calendar were included. No sample calculation was performed for this study. The volunteers were international level soccer players with at least five years of competitive experience in the sport. All the athletes agreed to participate in this investigation by signing a free and informed consent form. This study was conducted in accordance with the ethical principles outlined in the Declaration of Helsinki [22] and approved by the Research Ethics Committee in Research of the Santa Cecília University by CAEE: 83677724.2.0000.5513.

### 2.2. Research Design

The athletes were assessed in a competitive season at two distinct time points with six months’ difference between matches: one match in the first half of the season (Game 1), without the implementation of RELC and another match in the second half (Game 2), with RELC implementation. At the time of data collection, all athletes were in the same competitive phase of the season (mid-season) and completed at least six uninterrupted mesocycles of training under the same staff. Their conditioning was considered adequate based on full training participation, absence of injury, and stable internal load indicators, which confirmed physiological readiness for match demands. The key difference between the two matches was the implementation of load monitoring.

The monitoring of RELC was based on the metrics collected during both games, with the team’s physiologist tracking them in real-time via an antenna system (Catapult). These metrics were previously established by the physiology department. For both matches, the analysis considered the average of the top three individual values for each metric (total distance, sprint distance, number of sprints, accelerations, and decelerations), obtained through the Catapult One GPS tracking device (Catapult^®^, Melbourne, Austrália) with a 10 Hz frequency. Each athlete wore her own individual GPS device placed in a designated pocket of the uniform during matches. Monitoring was performed in real-time using the PlayerTek+ application, which allowed for live visualization of the metrics. After each session, the GPS data were downloaded via a synchronization base and analyzed using the Catapult Sync Tool software Vector 8 (S8), which generated performance reports.

### 2.3. Anthropometric Assessment

Anthropometric assessments were performed prior to the games, including body mass, height, and calculation of the body mass index (BMI); circumference; and skin folds. The measurements were conducted in the morning, with the athletes in a fasting state, wearing light clothing and being barefoot. All sports performance assessments were carried out in accordance with established standardized protocols. The athlete’s anthropometric data can be found in Table 1.

#### 2.3.1. Body Mass and Height

Body mass was measured as follows: the participant stepped onto the scale (Omron^®^ HBF-514C, Kyoto, Japan) with their back to the display, with their head pointing towards the Frankfurt plane, in an upright position and without moving. When stabilized, their body mass was measured. To assess height, the participants stood with their back to the stadiometer (Caprice Sanny^®^, São Bernardo do Campo, Brazil), also in an upright position and without moving, with their head pointing towards the Frankfurt plane. The participant took a full breath, and their height was measured before exhaling. After this procedure, the participant could return to breathing normally. Body mass and height values were used to calculate the BMI using the formula body mass (kg)/height^2^ (cm) and classified according to Bernal-Orozco et al. [23].

#### 2.3.2. Circumferences

A non-elastic measuring tape with millimeter precision (Sanny^®^, São Bernardo do Campo, Brazil) was used to measure body circumferences. The tape was passed through the anatomical point already determined at the beginning of the assessment. The athlete remained standing with her limbs relaxed, and her waist circumference was measured. The tape was passed through the point 3 times lightly and precisely without squeezing the region being assessed.

#### 2.3.3. Skinfolds

Skinfolds were measured with an adipometer calibrated according to the manufacturer’s instructions (Cescorf^®^ Neo Prime, Porto Alegre, Brasil) and the Jackson and Pollock [24] protocol was used, with the following 7 folds: chest, mid-axillary, triceps, subscapular, abdominal, supra-iliac, and mid-femoral. The procedures for taking the measurements included measurement on the right side of the body, with the thumb and index finger, the fold (subcutaneous tissue) highlighted, and the caliper positioned perpendicularly and approximately 1 cm below the fold. These measurements were taken 3 times at each anatomical point in an alternating manner. The result was calculated using the equation by Fagundes and Boscaini [25].

### 2.4. Study Design

#### 2.4.1. Load Control

Based on the metrics from each athlete’s three best matches of the season, an individual average was calculated for each parameter. These values were then used to plan weekly training loads [12]. The training week began on Game Day (GD), during which athletes were expected to reach 100% of their physical capacity. Subsequent days were labeled GD +1 (one day after the match), +2, and +3, and in the days leading up to the match, a countdown format was used (GD-1, -2, etc.). Each training session focused on a primary physical quality, aiming to prepare the athlete for supercompensation and optimal performance on Match Day [7,13]. Weekly distribution was calculated based on the average of the three best matches, with daily percentages assigned to each metric, percentages that are defined based on the periodization planned for the week. The load of these metrics close to game day did not exceed 45% of the maximum load to avoid causing late pain and fatigue, preserving the athlete so that she was fit for the game. Depending on the competition calendar, metrics were multiplied accordingly, and the sum of all daily metrics for the week was designed to match the total target load. We could define target load as each training session structured according to a predetermined target load, defined as the planned external load (volume × intensity) expected for that day based on the athlete’s profile, role, and proximity to competition (Game Day, GD).

A weekly sprint distance of 300 m was used as a reference target for players in attacking or wide positions, based on competitive demands. This target was adjusted weekly according to individual match exposure and acute/chronic workload ratios. For athletes who did not meet this value in matches, compensatory sprint drills were incorporated to complete the target load within the microcycle.

The acute/chronic workload ratio (ACWR) was used in line with the Gauss curve [26]. Acute load refers to the workload accumulated in the current week, while chronic load refers to the average over the past four weeks. The ACWR is calculated by dividing acute load by chronic load [12]. Ideal values range from 0.85 to 1.3. Exceeding or falling below this range increases the risk of musculoskeletal injury by up to 2.5 times [10,13,14].

#### 2.4.2. Using External Load Control During the Training

To implement the methodology of individualized external load management, training sessions were conducted daily using the Catapult One GPS tracking system. This allowed for real-time data analysis, enabling immediate adjustments in training volume and intensity to ensure compliance with daily load targets. If the external load deviated from the planned values (either above or below) training variables could be promptly modified to align with the individual’s prescribed load.

Load control planning was structured on a weekly basis, following evidence-based practices. We calculated each athlete’s individualized weekly target by multiplying a reference value defined as the average of their three highest official match performances (total distance covered, sprint distance, number of sprints, and number of accelerations and decelerations) by specific weekly weighting factors. Acceleration was defined as values greater than 1.5 m/s^2^, while deceleration was defined as values below this threshold, and the number of sprints was defined as movements exceeding 19 km/h. These factors were selected to allow for a controlled and progressive workload increase. For example, across a three-week mesocycle, we applied weighting factors of 2.8, 3.2, and 3.4, respectively, aiming for approximately 10% variation between weeks. These multipliers were used to estimate the total training volume, which was then distributed across weekly sessions, ensuring that load management remained individualized and context-specific for each athlete [27]. The weekly microcycle was structured using a reverse periodization approach, where the external load of the game (GD) served as the anchor point for planning the distribution of training volume and intensity throughout the week. The standard microcycle followed this pattern: GD-3 with the highest neuromechanical load (e.g., sprint exposure, HMLD), GD-2 with moderate aerobic demands, and GD-1 with a tapering session targeting ~45–50% of Match Day load. The weekly target load was then distributed proportionally, with total load adjusted based on match congestion, injury status, or recovery needs. To guide anaerobic-specific training, athletes were prescribed external loads that reached at least 90% of their individual Match-Day metrics (sprint distance, number of sprints, HMLD) in selected sessions. These thresholds were personalized based on each athlete’s most recent match data, ensuring that the training stimulus reflected the physiological demands required during competition.

Each metric was multiplied by its respective factor to modulate weekly intensity in accordance with the planned periodization model. These procedures were adapted from Ravé et al. [7], who emphasized the importance of remaining within safe ranges for acute and chronic workload ratios, thereby offering an integrated perspective on load evolution.

We also utilized Game Day (GD) and Match Day (MD) references to adjust planned loads. Typically, GD/MD+3 and +4 were the most physically demanding days. GD/MD–2 was used for high-speed and power-based stimuli, while GD/MD–1—falling within the 24 h prior to competition—was reserved for low-intensity activities to ensure optimal readiness and reduced fatigue on Match Day [7].

### 2.5. Statistical Analysis

Sample size calculation was performed using G*Power software 3.1.9.7, based on an effect size of 0.8, alpha of 0.05, and power of 0.80, indicating a minimum of 20 participants.

Data were expressed as mean ± standard deviation. One-way ANOVA was used to compare results, with significance set at *p* ≤ 0.05. Statistical analysis was performed using SPSS version 22.0. Prior to ANOVA, the Levene test was performed to confirm the assumption of homogeneity of variances.

## 3. Results

Table 1 presents the basic characteristics of the study participants, including the anthropometric data collected at the time of Game 1 and Game 2. The sample was classified as having a normal BMI, which corroborates the findings regarding the fat percentage, which were within the expected range for high-performance athletes, and the average waist circumference, which was also within the expected parameters. There were no statistical differences between the evaluations.

The data obtained revealed significant differences between the two assessment periods. There was a statistically significant increase in sprint distance (Game 1: 391 ± 244.2 m; Game 2: 450 ± 349.1 m; *p* = 0.045; d = 0.49) and in the total sprint count performed (Game 1: 14 ± 9.5; Game 2: 17 ± 12.5; *p* = 0.032; d = 0.35), indicating improved anaerobic performance following the implementation of RELC. The numerical increases were relatively small in the total distance, number of accelerations, and number of decelerations, not reaching statistical significance (*p* ≤ 0.05), and the corresponding effect sizes for these three variables were classified as trivial (total distance: d = 0.023; accelerations: d = 0.099; decelerations: d = 0.058), suggesting that the practical or clinical impact of these changes was minimal. These results highlight the finding that the most meaningful adaptations occurred in high-intensity actions (sprint distance and count), which showed small-to-moderate effect sizes (Table 2).

Total distance covered, accelerations, and decelerations did not show statistical significance between the two matches (*p* ≥ 0.05). However, an increase in the absolute means was observed in the second match, suggesting a trend of chronic adaptation to the volume and intensity of the prescribed load.

**Figure 1:**
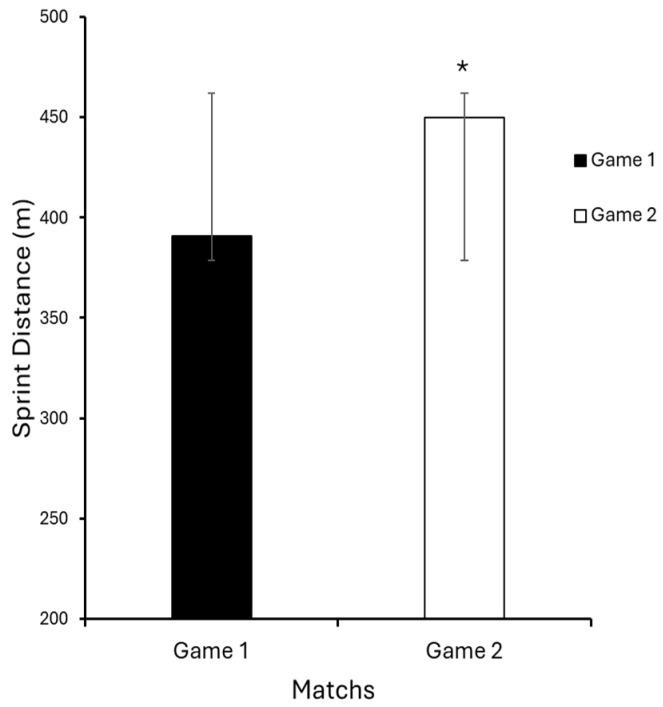
Sprint distance comparison between games. Data are presented as mean ± standard deviation. * *p* ≤ 0.05.

**Figure 2:**
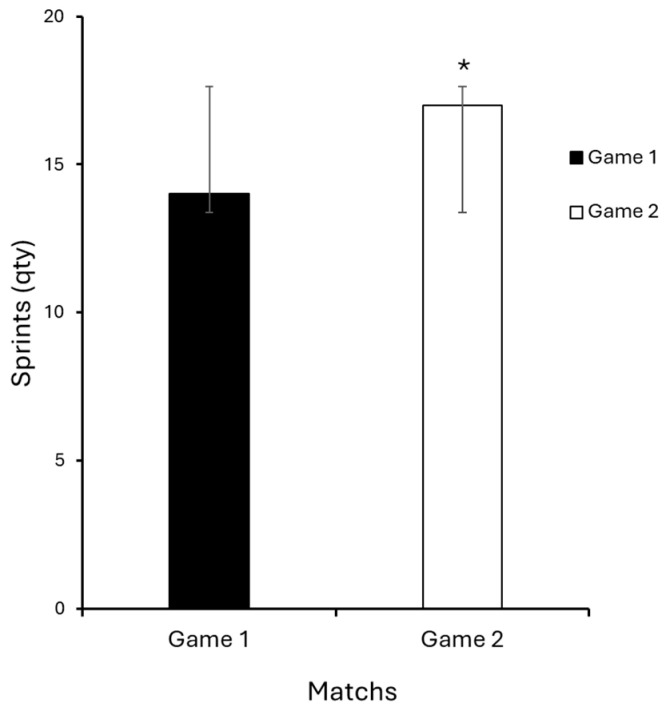
Sprint count (>19 km/h) comparison between games. Data are presented as mean ± standard deviation. * *p* ≤ 0.05.

Figure 1 illustrates the increase in sprint distance between matches, while Figure 2 highlights the rise in the number of sprints performed above 19 km/h, confirming the positive impact of REL on movement intensity. No statistically significant differences were found between the two matches for the variables total distance covered, number of accelerations, and decelerations (*p* ≥ 0.05). Nonetheless, an increase in the absolute mean values was observed in the second match, suggesting a trend of chronic adaptation to the volume and intensity of the prescribed load.

Figure 1 illustrates the increase in sprint distance between matches, while Figure 2 highlights the rise in the number of sprints performed above 19 km/h, confirming the positive impact of RELC on movement intensity.

## 4. Discussion

The results of this study align with the specialized literature, demonstrating that the individualization of external load, as suggested by Negrão and collaborators, represents an effective strategy for improving anaerobic performance in women’s soccer. The systematic application of RELC enabled fine adjustments in the prescription of physical qualities, maximizing the effects of supercompensation and optimizing functional readiness on MD [3]. Mathematical models were employed to ensure the feasibility and effectiveness of weekly external load prescriptions [15]. In each training session, a specific physical stimulus was assigned to each athlete, targeting a dominant physical attribute. This strategy prepares the athlete for future supercompensation and enhances performance on MD. These findings are consistent with our results, which show that load management effectively improved high-intensity performance metrics within group [13].

The significant increase in sprint distance and the number of sprints confirms the effectiveness of a data-driven physiological load distribution model, reinforcing the importance of periodization guided by objective metrics. As highlighted by Gómez-Piqueras and Alcaraz [10], the controlled introduction should be implemented through activities with a specific number of sprints, carefully designed in accordance with the weekly training load; a high-speed stimulus throughout the week (especially on MD–2 days) acts as an important mechanism for adaptive induction [15]. Our study demonstrated significant improvements in speed-related indicators, with sprint distance and the number of sprints (>19 km/h) showing the most notable gains. This suggests enhanced anaerobic capacity across the team, enabling players to sustain higher intensities during matches. These improvements are supported by previous research indicating that repeated high-intensity efforts and sprint performance are key predictors of success in elite soccer, particularly during offensive transitions and high-pressure defensive scenarios [28]. The improvements observed in sprint performance likely result from a combination of neuromechanical adaptations, including enhanced motor unit recruitment and increased rate of force development, alongside metabolic enhancements such as improved phosphocreatine resynthesis, glycolytic efficiency, and buffering capacity during high-intensity exercise. The increases in sprint distance and frequency indicate not only augmented neuromuscular function but also enhanced tactical readiness to perform explosive movements during crucial phases of competition. The capacity to repeatedly execute high-velocity actions throughout a match is strongly associated with greater influence on game outcomes and superior overall physical preparedness [29].

Additionally, sprint-related adaptations are closely linked to motor unit recruitment patterns and phosphocreatine (PCr) resynthesis efficiency, both fundamental components of anaerobic performance [29]. Training stimuli that enhance these systems, such as repeated sprint training and short high-speed exposures, support faster recovery between efforts and sustained peak velocities. Thus, the observed improvements in sprint metrics may reflect both neuromechanical adaptations and increased metabolic resilience to high-intensity demands [30,31].

The literature also highlights the relevance of individual maximum speed as a critical metric for defining training zones. According to Gómez-Piqueras and Alcaraz [10], using personalized high-speed thresholds (>90% of individual Vmax) provides greater accuracy in quantifying specific anaerobic stimuli. When applied to women’s soccer, this approach enables safer and more efficient training prescriptions that respect the biomechanical and physiological particularities of female athletes [10]. Moreover, the relevance of sprint training and progressive exposure to high-speed efforts was emphasized by de Villarreal et al. [32], who identified its direct relationship with improvements in power, movement economy, and neuromuscular readiness. In the present study, the observed improvement in sprint performance suggests that load control strategies may promote not only metabolic benefits but also neuromuscular enhancements. These findings open the possibility that individualized load management can contribute to broader performance adaptations, beyond those traditionally associated with metabolic conditioning. Further studies are warranted to explore these additional dimensions of adaptation. In this context, sprint efforts should be strategically distributed throughout the weekly microcycle, particularly during sessions that emphasize speed development. For these metrics to be used effectively, it is crucial to individualize each athlete’s maximum sprint speed rather than relying on fixed GPS thresholds (e.g., 23 km/h), which can lead to underestimation or overestimation of training stimuli based on individual capacity. Recent research supports the adoption of individualized velocity thresholds based on each athlete’s maximum sprint speed or maximal aerobic speed to improve training accuracy [33].

Relying on fixed velocity cutoffs may misclassify training intensities, especially in female athletes or those with divergent physical profiles. By personalizing speed zones, practitioners ensure that sprint-based sessions elicit appropriate neuromuscular demands, improving training specificity while minimizing the risks of undertraining or overload. Once load is individualized, sprint exposures can be purposefully integrated into the microcycle. The highest sprint load is typically scheduled for MD-2, although some models support including sprint work as early as MD-4. In addition to sprint count, monitoring sprint distance (in meters) per athlete is critical for precise load management. Importantly, applying this methodology requires careful consideration of total training volume to avoid excessive posterior thigh loading and to support optimal supercompensation [30,31,32]. Emerging models recommend progressively introducing sprint stimuli across the microcycle, with close attention to volume and intensity. According to Malone et al. [34], controlled sprint exposure on MD-4 and MD-2 helps maximize readiness while minimizing neuromuscular fatigue approaching MD, stimuli above 90% of MSS, distributed in controlled sprint exposures (<300 m total volume, short sprint sets, and rest intervals of 60–90 s).

Furthermore, it is essential to differentiate between sprint count and total sprint volume, as excessive repetition without sufficient recovery can degrade stimulus quality and hinder adaptation. Thus, both parameters should be balanced strategically within the week to optimize neuromuscular adaptation and minimize residual fatigue [35,36]. This strategy allowed for balancing neuromuscular stimulus and recovery by concentrating high-intensity exposures on GD–3 and surrounding them with lower-load sessions.

The lack of statistical significance in variables such as acceleration and deceleration should not be interpreted as a lack of progress, but rather as physiological stability in response to increased intensity—an indication of improved neuromuscular efficiency under repeated stimuli [19]. In line with the physiological principles proposed by Negrão, the adaptive response observed in this study confirms that periodization based on objective, individualized, and progressive data supports both athletic performance and long-term sports longevity [3]. Moreover, the concept of the “sweet spot” within the ACWR has been widely used to monitor and mitigate injury risk [37,38]. Athletes exposed to abrupt increases in weekly loads, especially in sprint-intensive sessions, are more prone to non-contact injuries. In contrast, those who maintain a consistently high chronic workload with controlled variability demonstrate better tissue tolerance and protection. Accordingly, embedding regular sprint exposures within a stable chronic load framework serves as both a performance enhancer and a cornerstone of prevention in elite soccer environments. Recent studies emphasize the importance of aligning external load strategies with individualized training responses to maximize both performance and recovery. Gabbett’s [39] “training-injury prevention paradox” suggests that athletes exposed to well-managed, high chronic loads are more protected than those experiencing irregular or insufficient workloads. This underscores the need for structured progressive overload guided by load monitoring to reduce tissue vulnerability.

Finally, these findings are consistent with the consensus outlined by Soligard et al. [40], which recommends tailoring training loads to match the athlete’s recovery and readiness. Such an approach supports both optimal performance and long-term athletic development [34].

Previous studies reinforce the role of individualized training in optimizing physical performance. For instance, Gonçalves et al. [41] demonstrated that tailoring training loads to individual profiles enhanced sprint capacity in elite female athletes, while Varjan et al. [42] found that individualization improved aerobic and anaerobic responses across a competitive season.

This study has some limitations that should be acknowledged. A larger number of matches comparing different load control strategies might have yielded more robust results. Additionally, the absence of a control group is a limitation, as the study design only included pre- and post-implementation assessments of load monitoring.

## 5. Conclusions

In conclusion, the individualized application of RELC demonstrated a positive contribution to the improvement of performance in professional women’s soccer players. Specifically, the observed increases in sprint distance and number of sprints suggest that tailoring training loads based on individual match data can optimize high-intensity outputs and promote meaningful physiological adaptations.

Practitioners aiming to implement this approach should begin by identifying each athlete’s three highest match performances across key external load metrics (total distance, sprint distance, accelerations, and decelerations) and use these as individualized reference values. Importantly, training sessions should be adjusted daily to ensure athletes are meeting, but not exceeding, their prescribed load, especially in the days leading up to competition.

This method allows coaching and performance staff to manage the workload with greater precision, enhancing both performance and recovery. The RELC model is particularly applicable in high-performance settings where player readiness and individualized programming are essential to sustaining competitive advantage throughout the season.

## Figures and Tables

**Table 1:** Anthropometric data before and after implementation of load control.

Variables	Game 1 (*n* = 27)	Game 2 (*n* = 27)
Body mass (kg)	64.1 ± 12.5	66.3 ± 14.3
Height (m)	1.68 ± 0.1	1.68 ± 0.1
BMI (kg/m^2^)	23.0 ± 4	23.2 ± 5.7
Body fat (%)	14.6 ± 3.2	13.9 ± 3.8
Waist circumference (cm)	74.2 ± 9	73.8 ± 9

Data expressed as mean and standard deviation.

**Table 2:** Comparison of GPS metrics between Game 1 and Game 2.

Metric	Game 1	Game 2	*p*-Value	Effect Size (d)
Total distance (km)	8.6 ± 3.7	8.7 ± 4.9	≥0.05	0.023
Sprint distance (m)	391 ± 244.2	450 ± 349.1 *	0.045	0.49
Sprint count (qty)	14 ± 9.5	17 ± 12.5 *	0.032	0.35
Accelerations (qty)	140 ± 62	147 ± 78	≥0.05	0.099
Decelerations (qty)	149 ± 76	154 ± 95	≥0.05	0.058

Km: kilometers, m: meters, qty: quantity. * Statistically significant differences (*p* ≤ 0.05). Data are expressed as mean and standard deviation.

## Data Availability

The data generated during the current study are available from the corresponding author on reasonable request. The data are not publicy available due to Protection of research participants’ data.

## Abbreviations

The following abbreviations are used in this manuscript:

RELC	Relative external load control
GD	Game day
MD	Match day
ACWR	Acute/chronic workload ratio
PCr	Phosphocreatine
HIIT	High-intensity interval training
